# Enhanced photocatalytic activity of Ag–ZnO hybrid plasmonic nanostructures prepared by a facile wet chemical method

**DOI:** 10.3762/bjnano.5.75

**Published:** 2014-05-15

**Authors:** Sini Kuriakose, Vandana Choudhary, Biswarup Satpati, Satyabrata Mohapatra

**Affiliations:** 1 School of Basic and Applied Sciences, Guru Gobind Singh Indraprastha University, Dwarka, New Delhi 110078, India; 2 Saha Institute of Nuclear Physics, 1/AF Bidhannagar, Kolkata 700064, India

**Keywords:** Ag–ZnO, hybrid plasmonic nanostructures, photocatalysis

## Abstract

We report the synthesis of Ag–ZnO hybrid plasmonic nanostructures with enhanced photocatalytic activity by a facile wet-chemical method. The structural, optical, plasmonic and photocatalytic properties of the Ag–ZnO hybrid nanostructures were studied by X-ray diffraction (XRD), field emission scanning electron microscopy (FESEM), transmission electron microscopy (TEM), photoluminescence (PL) and UV–visible absorption spectroscopy. The effects of citrate concentration and Ag nanoparticle loading on the photocatalytic activity of Ag–ZnO hybrid nanostructures towards sun-light driven degradation of methylene blue (MB) have been investigated. Increase in citrate concentration has been found to result in the formation of nanodisk-like structures, due to citrate-assisted oriented attachment of ZnO nanoparticles. The decoration of ZnO nanostructures with Ag nanoparticles resulted in a significant enhancement of the photocatalytic degradation efficiency, which has been found to increase with the extent of Ag nanoparticle loading.

## Introduction

The removal of hazardous materials such as dyes and organic compounds from waste water has attracted ever increasing attention over the years. Semiconductor photocatalysis is one of the most important technologies used for the complete mineralization of a wide range of organic dyes and toxic chemicals. ZnO, a wide band gap semiconductor with large excitonic binding energy is suitable for diverse applications including UV lasers [1], field effect transistors [2], dye sensitized solar cells [3–4], surface enhanced Raman spectroscopy (SERS) [5] and biomedical applications [6–10]. ZnO nanostructures are promising photocatalysts because of their high quantum efficiency, high redox potential, superior physical and chemical stability, non-toxicity and low cost [11–16]. However, ZnO nanostructures suffer from drawbacks such as a high electron–hole recombination rate and the inefficient utilization of sun light, which limit their photocatalytic activity [17–18]. Several attempts have been made to improve the photocatalytic efficiency of ZnO by decreasing the rate of recombination of electrons and holes by surface modification with noble metal nanoparticles [19–24]. Surface modification of ZnO nanostructures with noble metal nanoparticles improves the photocatalytic efficiency, since the noble metal–ZnO system has two distinct features. Firstly, noble metal–ZnO contacts result in a Schottky junction, which creates an internal electric field close to the interface causing the photogenerated electrons and holes to move in different directions, which results in a decrease of their recombination rate [19]. Secondly, noble metal nanoparticles on ZnO exhibit localized surface plasmon resonance (LSPR) absorption of light which can have significant impact on semiconductor photocatalysis. The LSPR wavelength of noble metal nanoparticles can be tuned from near UV to the visible region by controlling their size, shape, inter-particle spacing and surrounding medium [25]. In case of ZnO modified with noble metal nanoparticles, LSPR absorption can lead to enhanced utilization of UV–visible light as compared to pure ZnO [26–28]. Silver nanoparticles decorated ZnO nanostructures of various morphology have shown considerable increase in the photocatalytic efficiency for the degradation of organic dyes [19,23,29–34]. Xie et al. [23] have shown that Ag loading on ZnO nanostructures improves its photostability and enhances the photocatalytic activity due to increased efficiency for separation of photogenerated electrons and holes. It has been shown that Ag–ZnO nanostructures take 80 min for the complete photocatalytic degradation of 0.2 μM crystal violet dye under UV irradiation. Liu et al. [30] have studied the effects of Ag loading on ZnO on the photocatalytic degradation of rhodamine B (RhB) and showed that the degradation of RhB over pure Ag nanowires was negligible as compared to ZnO, the degradation efficiency of which further was increased due to the decoration with Ag nanoparticles. Deng et al. [19] fabricated Ag nanoparticles decorated ZnO microrods, by photoreduction of Ag ions onto the surface of the ZnO microrods prepared through a solvothermal-assisted method, which showed enhanced sun light active photocatalytic activity. In this paper, we report the synthesis of Ag–ZnO hybrid plasmonic nanostructures by a two-step facile wet chemical method involving the trisodium citrate assisted photoreduction of Ag ions onto the surface of ZnO nanostructures, prepared by a facile wet chemical method. The effects of citrate concentration and Ag nanoparticle loading on the photocatalytic activity of Ag–ZnO hybrid plasmonic nanostructures towards sun-light driven degradation of methylene blue (MB) dye have been investigated.

## Results and Discussion

### Morphology and crystal structure

**Figure 1:**
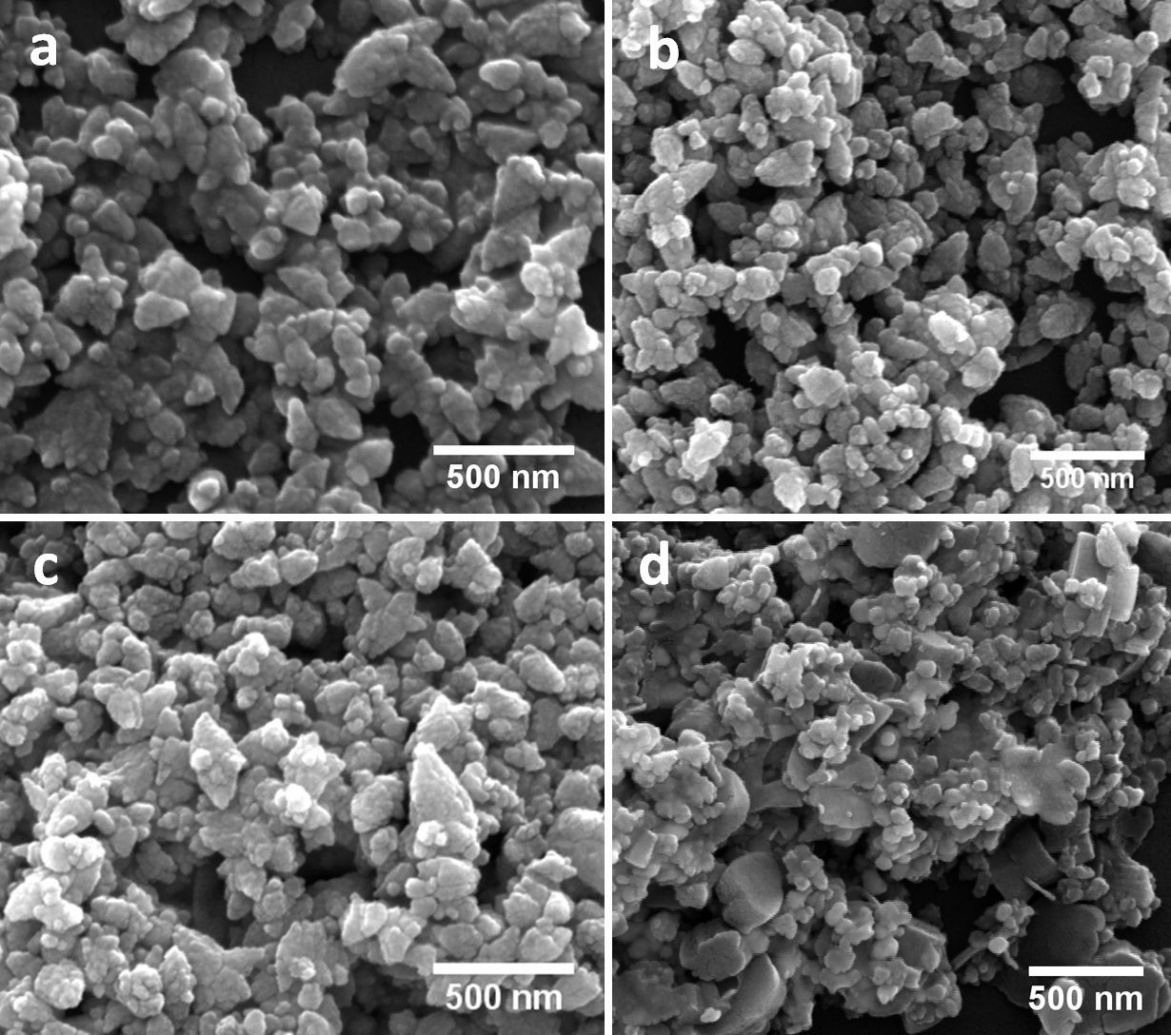
FESEM images of as-synthesized samples (a) PZ, (b) AZ21, (c) AZ410 and (d) AZ510 showing the effect of citrate concentration on the morphology of the samples.

FESEM images of as-synthesized ZnO and Ag–ZnO samples with varying citrate concentrations for different [Ag^+^]/[citrate] ratios are shown in Figure 1. The presence of aggregates of ZnO nanoparticles of anisotropic shapes can be seen in the FESEM image (Figure 1a) of the pristine ZnO sample. Addition of citrate at 0.2 mM concentration resulted in an increased aggregation of the nanoparticles, as shown in Figure 1b. As the citrate concentration is increased to 10 mM, the oriented attachment of the aggregated nanoparticles resulted in complex nanostructures (Figure 1c). In Figure 1d we show the FESEM image of Ag–ZnO sample AZ510, which was prepared by using citrate a concentration of 20 mM and a AgNO_3_ concentration of 2 mM. It can be clearly seen that as the citrate concentration is increased to 20 mM, nanodisk-like structures formed due to oriented attachment of aggregating nanoparticles. It is evident from the FESEM images that above a threshold concentration trisodium citrate assists in the oriented attachment of ZnO nanoparticles and leads to the formation of nanodisk-like structures, even at room temperature. Cao et al. [35] have studied the effects of citrate on the morphology of the ZnO nanostructures. It has been shown that citrate ions bind to the ZnO(0001) surface through the –COOH and –OH groups and suppress the growth along the <0001> direction. Thus, growth proceeds sideways, which leads to the formation of nanodisk-like structures. Our FESM results showing formation of nanodisk-like structures at higher citrate concentration go in line with this.

**Figure 2:**
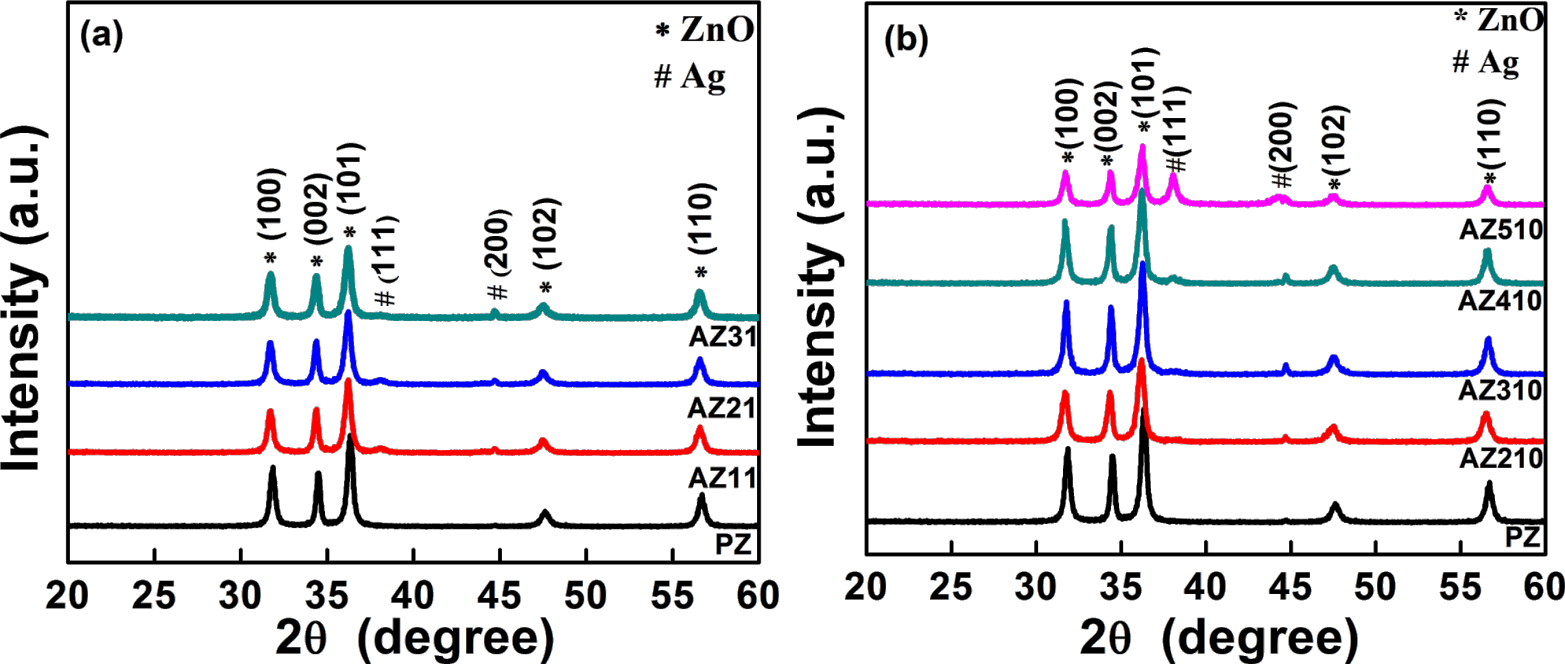
XRD patterns of as-synthesized ZnO and Ag–ZnO samples prepared with varying AgNO_3_ concentrations and different [Ag^+^]/[citrate] ratios (a) 1:1, (b) 1:10.

Figure 2 shows the XRD patterns of as-synthesized ZnO and Ag–ZnO samples (sample nomenclature shown in Table 1, see section Experimental) prepared with varying [Ag^+^]/[citrate] ratios and AgNO_3_ concentrations. The observed peaks can be well indexed to the hexagonal wurtzite structure of bulk crystalline ZnO [JCPDS no. 36-1451] and the face centred cubic structure of Ag [JCPDS card no. 04-0783]. Appearance of Ag peaks in the diffraction patterns clearly indicates the formation of crystalline Ag nanoparticles by photoreduction onto ZnO nanostructures. No extra peaks related to any impurity or silver oxides were observed, which confirms that the as-synthesized products are pure wurtzite ZnO and Ag–ZnO hybrid nanostructures. The average crystallite size of the ZnO nanoparticles was estimated to be about 20 nm, while that of Ag nanoparticles varied from 8 to 20 nm in different as-synthesized Ag–ZnO samples.

**Figure 3:**
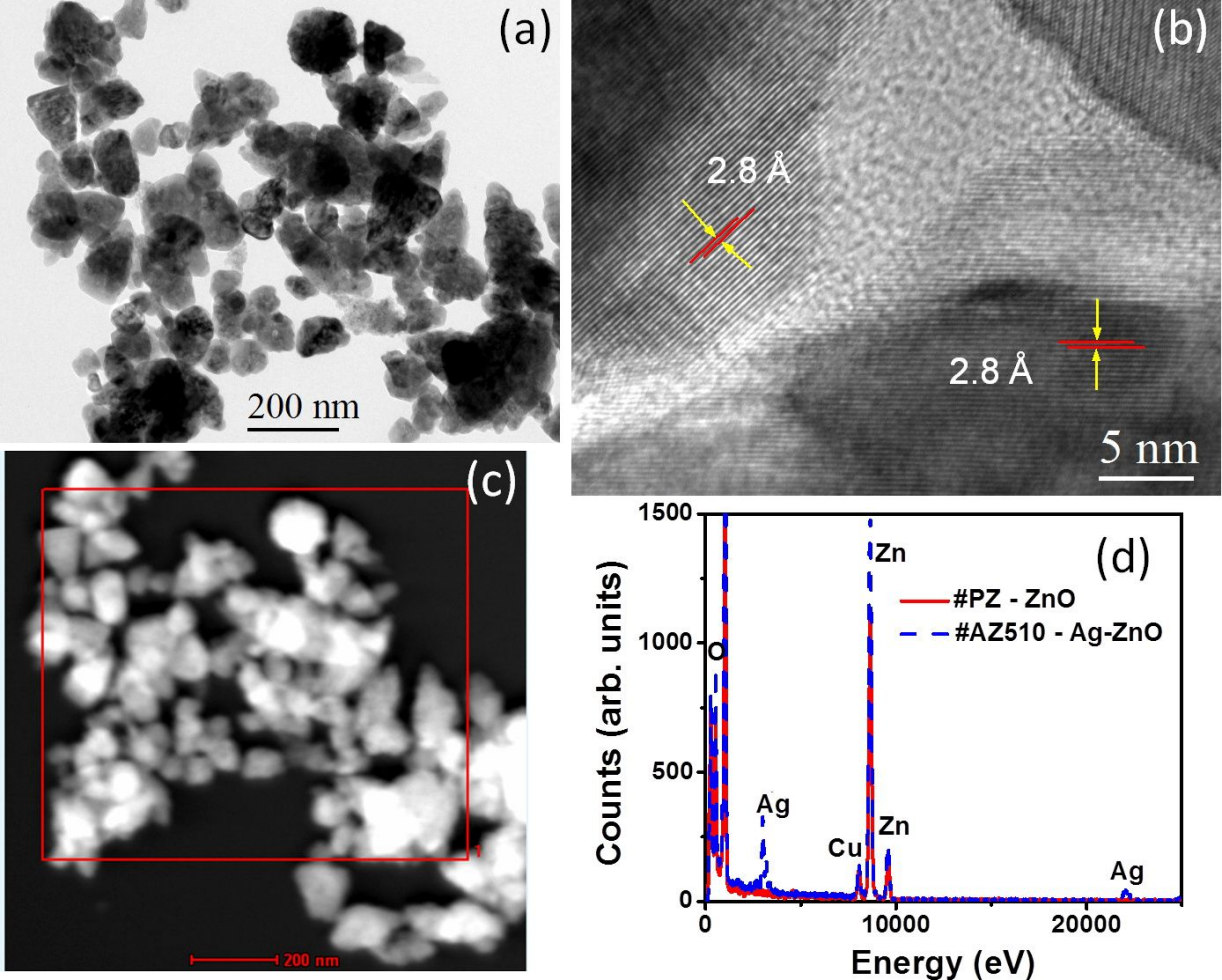
(a) Low-magnification TEM image of ZnO nanostructures in sample PZ. (b) HRTEM image showing lattice fringes. (c) STEM-HAADF image from the same area of TEM image. (d) EDX spectra from a region marked by area 1 in (c) and from area 2 in Figure 4c.

**Figure 4:**
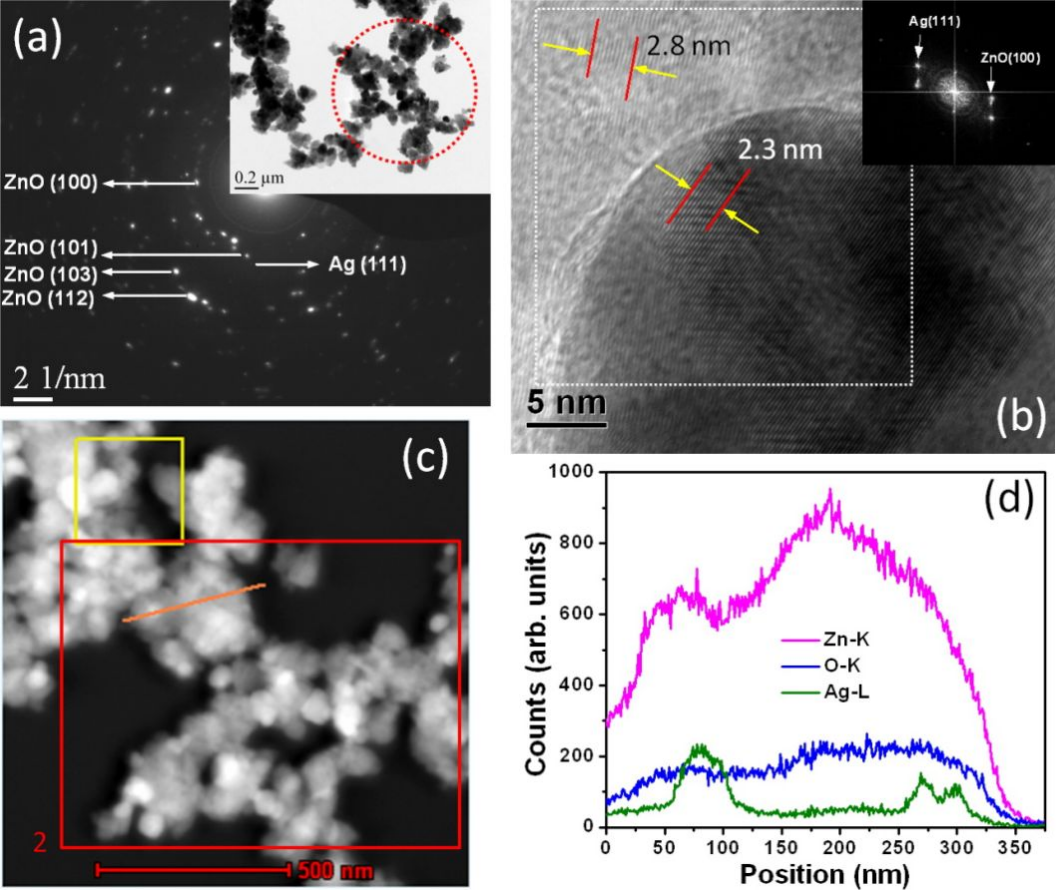
(a) Selected area diffraction pattern from Ag–ZnO hybrid nanostructures and in the inset low magnification TEM image of sample AZ510. (b) HRTEM image showing lattice fringes and in the inset FFT from a region marked by dotted box. (c) STEM-HAADF image from the same area of the TEM image. (d) EDX line profile from a region marked by line in (c) showing the distribution of different constituent elements across the nanostructures.

In-depth structural information was further obtained using TEM studies. From a low-magnification TEM image (Figure 3a) of sample PZ, the presence of ZnO nanostructures of anisotropic shapes can be clearly seen. Higher magnification images revealed that these anisotropic nanostructures consist of smaller nanoparticles and are formed through aggregation. TEM images of AZ510 sample revealed the presence of anisotropic nanostructures decorated with nanoparticles. HRTEM study of these decorating nanoparticles confirmed them to be of Ag. Figure 4b shows the selected area diffraction (SAD) pattern from a region marked by a dotted circle. The SAD pattern shows concentric rings consisting of distinct spots, which is because of the presence of many small crystals and suggests the crystalline nature of heterostructures. The SAD pattern further confirms the formation of crystalline hexagonal phase of Ag–ZnO hybrid nanostructures. The high-resolution TEM image of ZnO nanostructures in Figure 3b clearly shows lattice fringes and the measured lattice spacing is 2.8 Å. The HRTEM image of of Ag–ZnO hybrid nanostructures shown in Figure 4b reveals lattice fringes of 2.3 Å and 2.8 Å, which correspond to the (111) and (100) interplanar spacing (*d-*spacings) of Ag and ZnO, respectively. Some of the measured *d-*spacings from the SAD pattern of Figure 4a are 2.84 Å, 2.50 Å, 1.49 Å, and 1.39 Å and these may be assigned as (100), (101), (103) and (112) interplanar spacing of hexagonal ZnO (*d*(100), *d*(101), *d*(103) and *d*(112) of ZnO are 2.81 Å, 2.47 Å, 1.47 Å, 1.37 Å, respectively) [JCPDS 36-1451]. In the SAD pattern there are also spots corresponding to Ag and one of them is marked in Figure 4a.

STEM-HAADF analysis was carried out to investigate the chemical composition of the Ag–ZnO hybrid nanostructures. STEM-HAADF analysis provides the *Z*-contrast image, where the intensity of scattered electrons is proportional to the square of the atomic number *Z*. Figure 3c shows the STEM-HAADF image of ZnO nanostructures in sample PZ. Energy dispersive X-ray spectroscopy (EDX) data from the regions marked by area 1 in Figure 3c and area 2 in Figure 4c is plotted in Figure 3d for ZnO and Ag–ZnO. The C and Cu signals in the EDX spectra are due to carbon-coated copper grid. The drift corrected EDX line profile was used to obtain the spatial distributions of the atomic contents across the Ag–ZnO nanostructures. Figure 4d shows the EDX profiles for Zn, O and Ag across the line marked in Figure 4c.

**Figure 5:**
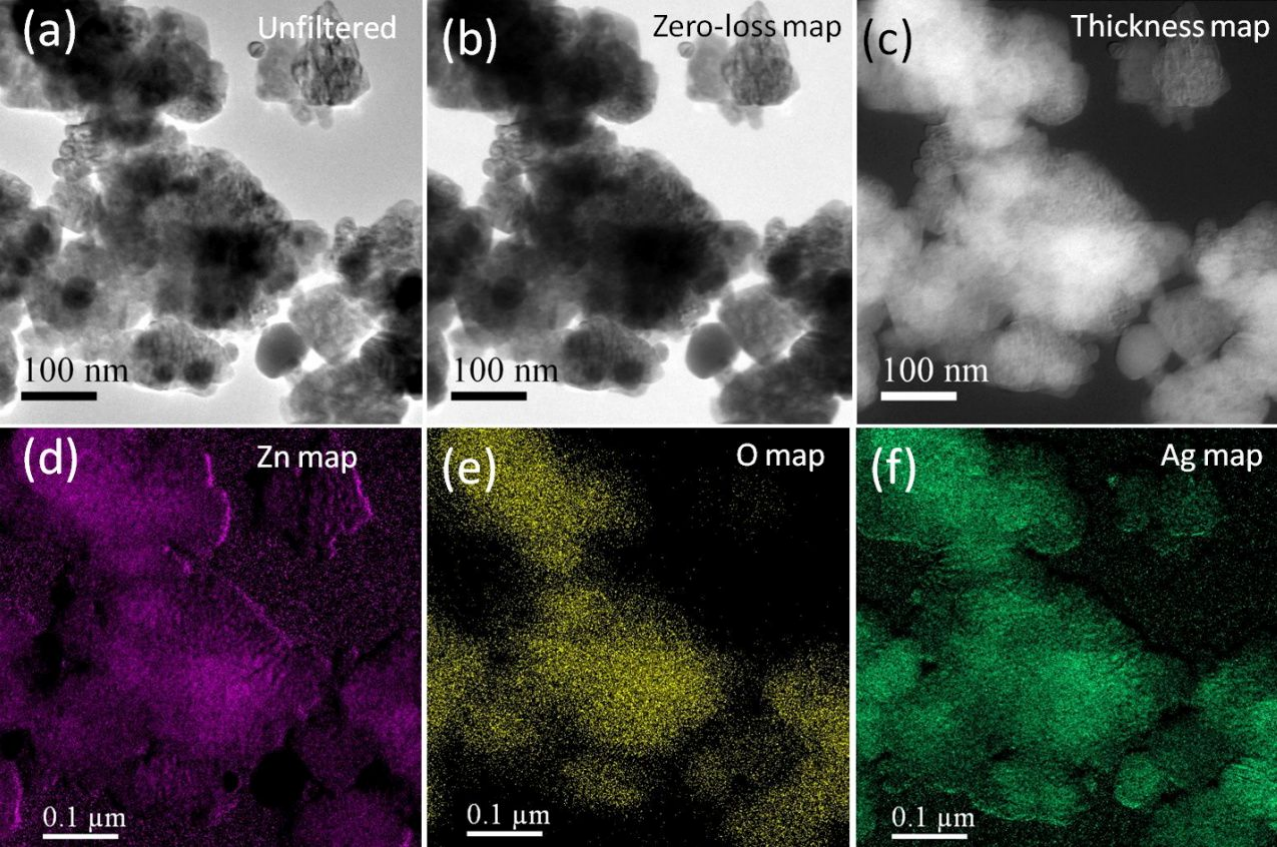
EFTEM images taken from the same area of a TEM image indicating the locations of different atoms across the nanostructure in sample AZ510. (a) Unfiltered image. (b) Zero-loss image. (c) Relative thickness map. (d) Chemical map of Zn (pink). (e) Chemical map of O (yellow). (f) Chemical map of Ag (green).

Figure 5 show the elemental mapping using EFTEM for obtaining the distributions of Zn, O and Ag atoms in the Ag–ZnO hybrid nanostructures. Chemical maps from Zn M (87 eV), O K (532 eV) and Ag N (56 eV) edges were obtained using the jump-ratio method by acquiring two images (one post-edge and one pre-edge), respectively, to extract the background, with an energy slit of 8 eV for Zn, 30 eV for O and 2 eV for Ag. The observed EFTEM images confirmed the decoration of ZnO nanostructures with Ag nanoparticles.

### Optical absorption and photoluminescence

**Figure 6:**
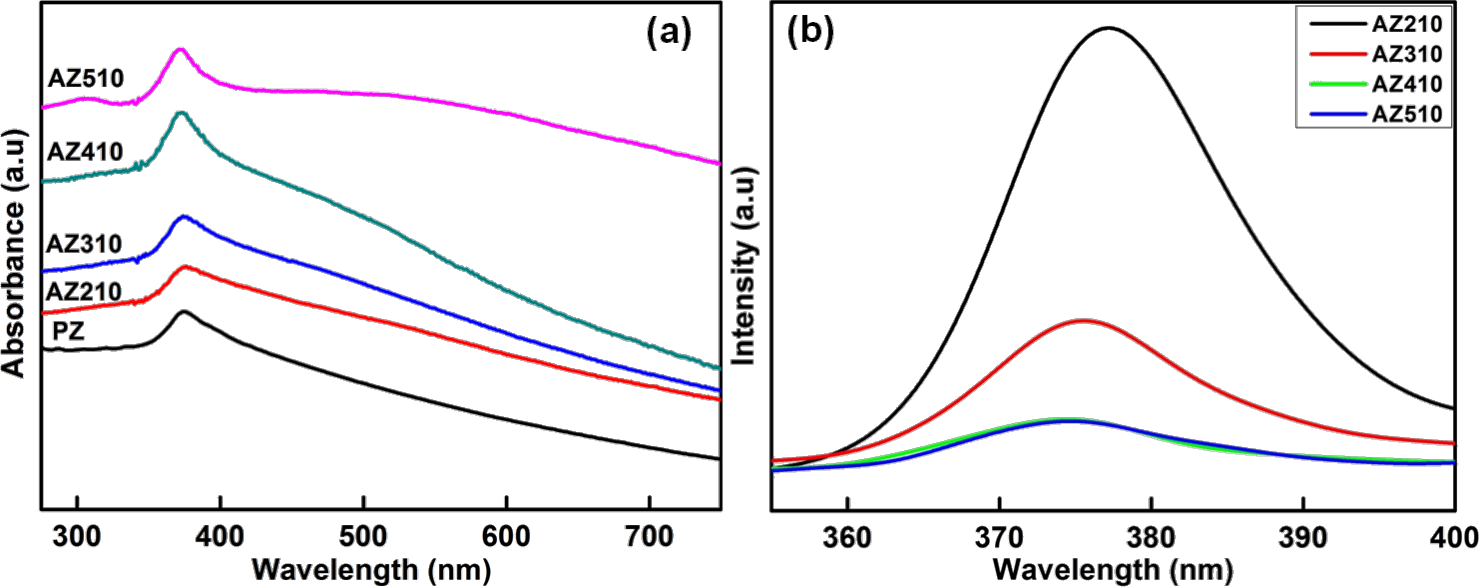
(a) UV-visible absorption spectra of samples AZ210, AZ310, AZ410 and AZ510 with varying Ag concentrations and [Ag]/[citrate] ratio of 1:10 and (b) corresponding room temperature PL spectra of these samples.

The UV–visible absorption spectra of samples with varying Ag concentration are shown in Figure 6a. It can be seen that Ag–ZnO samples exhibit two prominent absorption peaks. The first peak around 375 nm is attributed to the excitonic absorption peak of ZnO nanostructures. A weak and broad band around 480 nm has been found to emerge as the Ag concentration is increased. This band has been found to red shift, broaden and increase in intensity with increase in the extent of Ag nanoparticles loading onto ZnO nanostructures. It can be clearly seen that the sample AZ510 with the highest Ag concentration (2 mM) and citrate concentration (20 mM) exhibits a very broad band around 500 nm with much higher intensity as compared to other samples.

The observed broad bands around 480–500 nm are the characteristic SPR peak of Ag nanoparticles [23] and confirm the formation of Ag nanoparticles by photoreduction onto ZnO nanostructures. It should be pointed out here that Ag nanoparticles prepared by citrate-assisted reduction in aqueous solution shows SPR peaks around 400 nm. It is known that the SPR wavelength of noble metal nanoparticles can be tuned by tailoring the size, shape, inter-particle spacing and the surrounding medium [25]. Deposition of Ag nanoparticles onto ZnO nanostructures with higher refractive index leads to red shift in SPR. In addition, reduced inter-particle spacing due to increased Ag loading is expected to contribute to the observed red shift and significant broadening of the SPR peak, due to stronger electromagnetic coupling within the Ag nanoparticles deposited onto ZnO nanostructures.

The room-temperature PL spectra of Ag–ZnO samples with varying Ag concentrations are shown in Figure 6b. The peak at 375 nm is the near band edge emission peak of ZnO [19]. It is observed that the intensity of UV emission decreased with an increase in the Ag content of the samples. The decrease of intensity in UV region clearly indicates that the recombination of electrons and holes is suppressed [36]. The Ag nanoparticles deposited on the ZnO nanostructures act as sinks for the photogenerated electrons and hence result in the suppression of their recombination with the holes. Increase in Ag nanoparticle loading onto ZnO nanostructures leads to an efficient suppression of recombination of photogenerated electrons and holes, which, in turn, improves the photocatalytic efficiency.

### Mechanism of citrate assisted growth of nanodisks

The formation of ZnO nanoparticles from aqueous solutions of zinc nitrate and KOH involves the following reactions [37]:


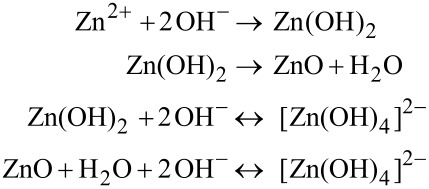


The concentration of KOH is an important factor in deciding the morphology of the ZnO nanostructures that are formed. The addition of aqueous KOH into Zn salt solution leads to formation of white precipitates of Zn(OH)_2_, which decompose to form ZnO nuclei. Depending on the Zn^2+^ concentration and synthesis conditions, ZnO nuclei grow into nanoparticles. In the presence of excess OH^−^ ions (because of a higher KOH concentration) [Zn(OH)_4_]^2−^ ions form, which help in formation of aggregates of ZnO nanoparticles. As seen from the FESEM results (Figure 1), the amount of trisodium citrate has a significant effect on the morphology of the Ag–ZnO nanostructures. When the concentration of citrate is 1 mM, there is almost no change in the morphology of the sample as compared to that of pristine ZnO. However, when the citrate concentration was increased to 5 mM oriented attachment of the nanoparticles led to formation of complex shaped nanostructures. With the further increase of citrate concentration to 20 mM, nanodisk-like structures formed. Citrate ions with –COOH and –OH groups preferentially get adsorbed on the (0001) surface and prevent the accumulation of growth units on the (0001) surface. Because of this the growth of ZnO crystallites occurs along the six symmetric directions, producing ZnO nanodisks [38]. Thus the morphology of ZnO nanostructures can be easily altered by using trisodium citrate [39]. Detailed studies on the effects of trisodium citrate on the shape evolution of ZnO nanostructures will be reported elsewhere.

### Photocatalytic studies

**Figure 7:**
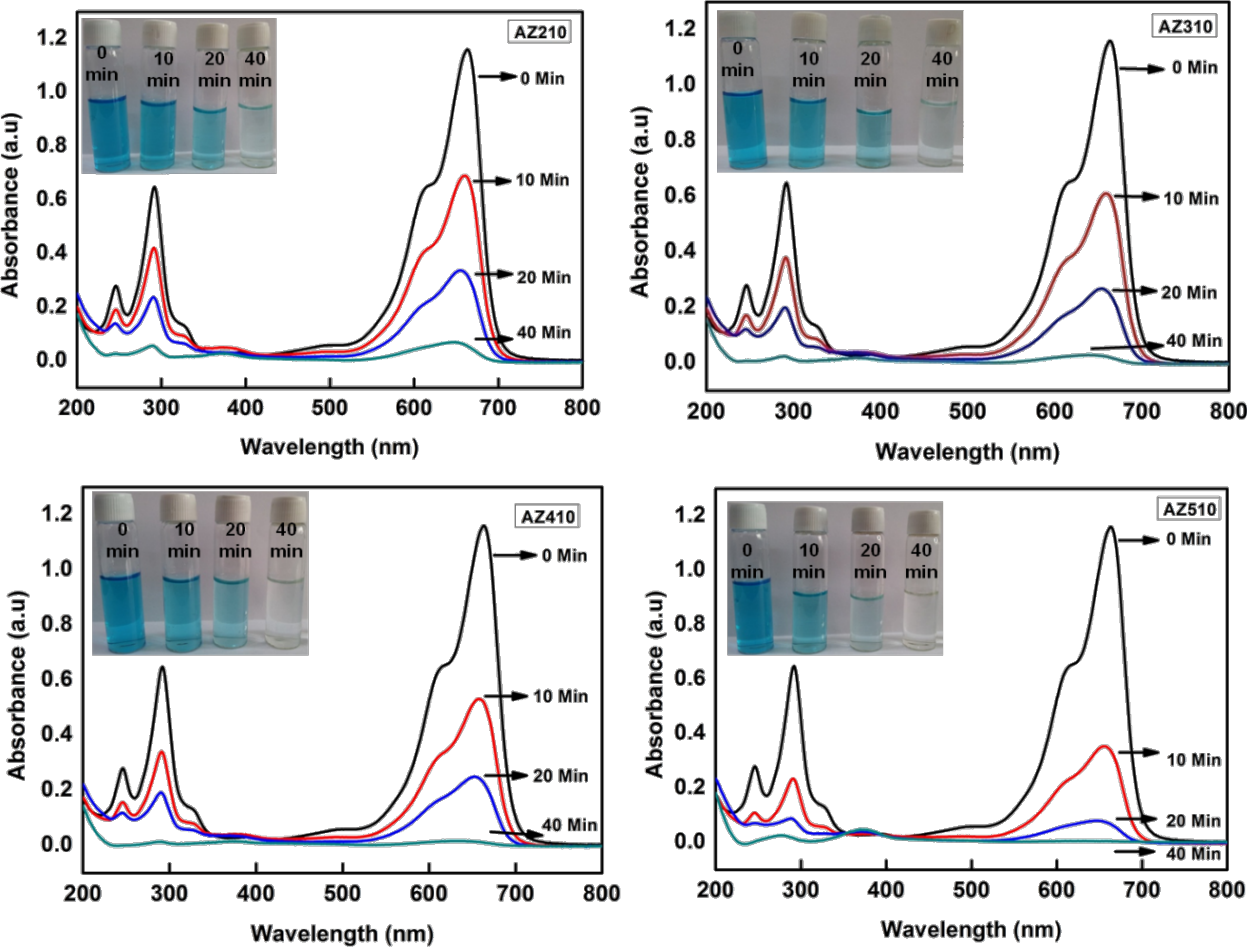
UV–visible absorption spectra showing the temporal evolution of the degradation of MB upon sun-light irradiation using Ag–ZnO samples AZ210, AZ310, AZ410 and AZ510, prepared with different AgNO_3_ concentrations and a [Ag^+^]/[citrate] ratio of 1: 10, as photocatalysts.

Figure 7 shows the UV–visible absorption spectra of 10 μM MB aqueous solutions with different photocatalysts AZ210, AZ310, AZ410 and AZ510 following the irradiation with sun light for different durations of time. The characteristic absorption peak of MB at 664 nm is monitored as a function of sun-light exposure time. From Figure 7, it is clear that Ag–ZnO samples with higher Ag content lead to more efficient degradation of MB for the same exposure time. It can also be clearly seen that the photocatalytic efficiency is highest for AZ510, which has the maximum Ag nanoparticles loading and nanodisk-like structures, formed because of the higher citrate concentration.

**Figure 8:**
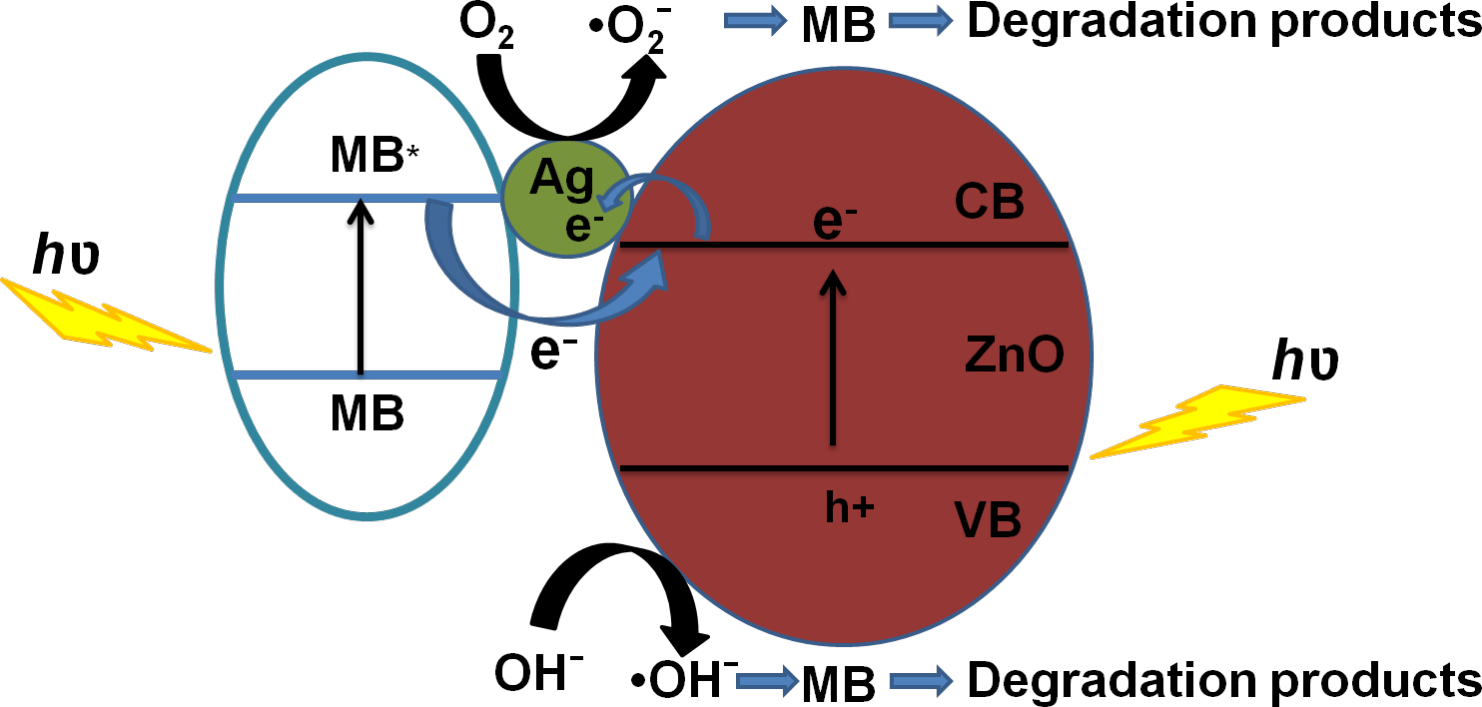
Schematic band diagram of Ag–ZnO hybrid nanostructure showing the charge redistribution processes that lead to the photocatalytic degradation of MB dye.

The mechanisms underlying the enhanced photocatalytic activity of Ag–ZnO hybrid plasmonic nanostructures towards the degradation of MB can be understood as follows: The SPR of Ag nanoparticles helps in extending the light absorption of ZnO from near UV to the visible region, leading to an improved sun-light utilization efficiency. In addition, decoration with Ag nanoparticles significantly improves the charge separation in ZnO. When ZnO absorbs photons of energy greater than or equal to its band gap, electrons are promoted from its valence band to conduction band, creating an equal number of holes in the valence band. Since the energy level of conduction band of ZnO is higher than the Fermi level of Ag–ZnO hybrid structure, electrons flow from ZnO nanostructures to Ag nanoparticles. This way Ag nanoparticles act as efficient sinks for the photogenerated electrons, preventing their recombination with holes. This process, known as the direct electron transfer from semiconductor to the plasmonic nanostructures, is dependent on the alignment of electronic band structure of the noble metal and semiconductor. Furthermore, irradiation with sun light leads to the excitation of MB dye molecules adsorbed onto the ZnO nanostructures. The photoexcited MB molecules transfer electrons into the conduction band of ZnO [40]. The photogenerated electrons created by the above mentioned processes react with dissolved O_2_ molecules forming superoxide anion radicals, while holes react with H_2_O leading to the formation of hydroxyl radicals, both of which cause the degradation of the MB dye. These reactions can be summarized as follows [29–30] and are schematically illustrated in Figure 8.


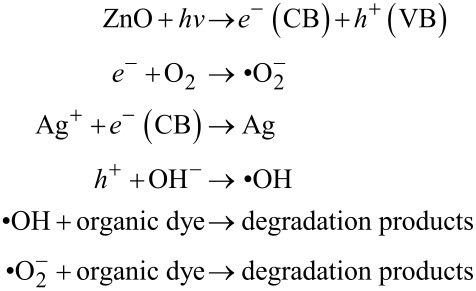


Yin et al. [41] prepared nanocomposites with Ag nanoparticle decorated ZnO nanorods with a core–shell structure by seed-mediated method. They have shown that Ag–ZnO is a better photocatalyst than ZnO because, firstly, the nanocomposites have a larger surface area as compared to ZnO, which leads to enhanced adsorption of dye. Secondly, due to the decoration of ZnO with Ag nanoparticles, the recombination of electrons and holes are inhibited. Gao et al. [42] synthesized Ag–ZnO nanocomposites by a biomolecule assisted hydrothermal method and studied their photocatalytic properties. They concluded that Ag nanoparticles improve the separation of electron and holes by acting as electron sinks. In our case, the photocatalytic efficiency is highest for sample AZ510, with nanodisk-like structures having higher surface area, and maximum Ag nanoparticle loading. The BET surface area of the pristine sample PZ and the sample AZ510 prepared with the highest citrate concentration were found out to be 13.5 and 15.9 m^2^·g^−1^, respectively.

**Figure 9:**
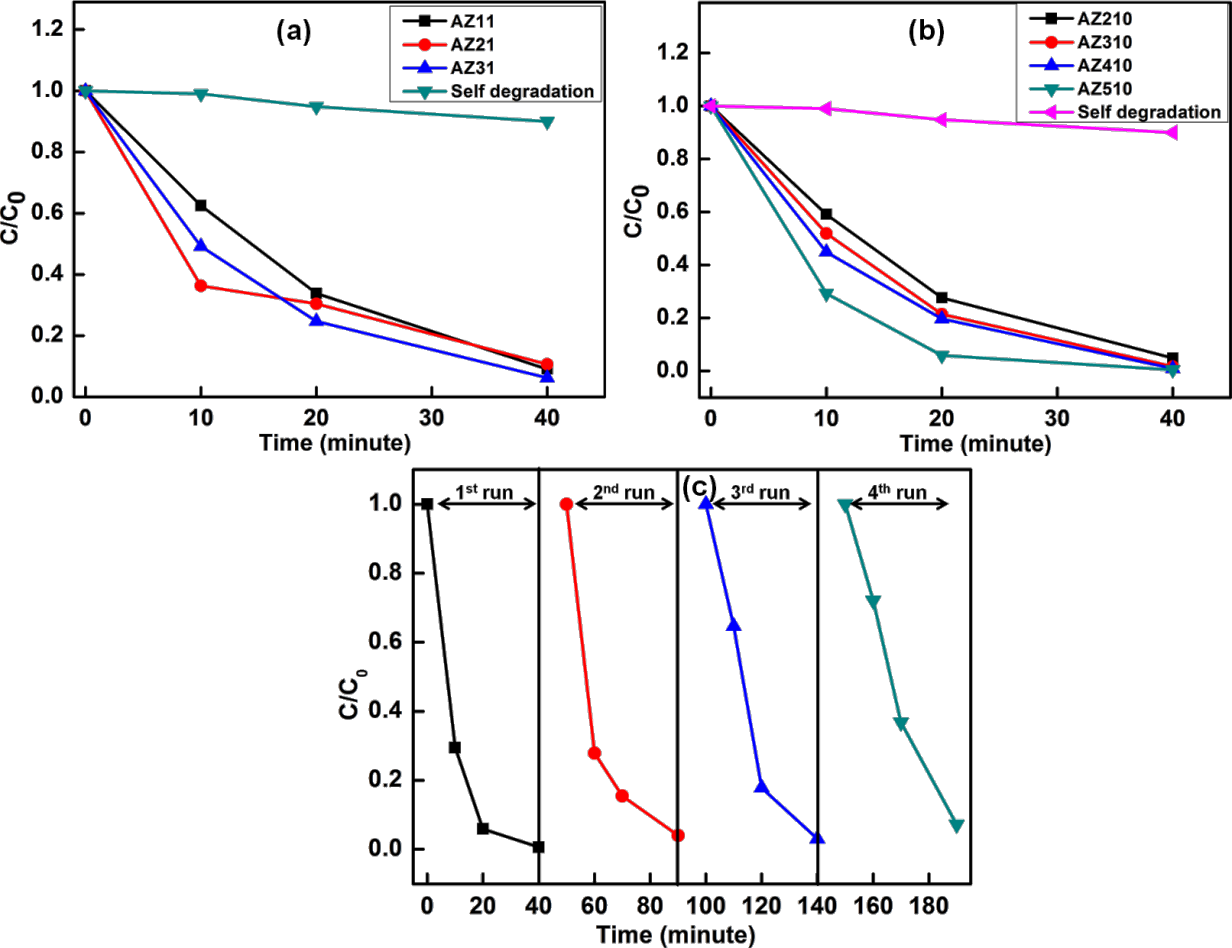
(a,b) Kinetics of MB photodegradation by Ag–ZnO hybrid plasmonic nanostructures with different Ag nanoparticle loading for different [Ag^+^]/[citrate] concentrations. (c) Repetitive test for AZ510 photocatalyst for four runs.

Figure 9 shows the kinetics of MB degradation by using different Ag–ZnO hybrid nanostructures as photocatalysts under sun-light exposure. Figure 9a,b show the kinetics of MB degradation for photocatalysts with different Ag nanoparticles loading by using different [Ag^+^]/[citrate] ratios 1:1 and 1:10. It can be seen that pristine ZnO nanostructures degraded only 52% of MB following 20 min of sun-light exposure, whereas all the Ag–ZnO hybrid plasmonic nanostructures led to enhanced photodegradation for the same exposure time. Among the various Ag–ZnO photocatalysts used, sample AZ510 exhibited the highest photocatalytic efficiency of 94% for the same exposure time of 20 min. Figure 9c shows the results of repetitive tests of the photocatalytic activity of AZ510 sample for four runs. It can be clearly seen that the efficiency of the photocatalyst remains high even after four runs.

The effects of citrate concentration and Ag loading on the photocatalytic efficiency can be summarized as follows. It can be clearly seen from that for the same citrate concentration, the photocatalytic efficiency increases with increasing Ag loading. Also, for the same Ag concentration, an increase in citrate concentration has been found to result in an increased efficiency of the photodegradation of MB. This clearly indicates that increase in the photocatalytic efficiency of the synthesized Ag–ZnO hybrid plasmonic nanostructures is mainly due to citrate-assisted morphological changes and changes in plasmonic coupling due to different level of Ag nanoparticle decoration. Beyond a threshold concentration, citrate plays an important role in changing the morphology of ZnO nanostructures so that it has a larger surface area as compared to the pristine sample. The sample AZ510, prepared with highest citrate concentration and with maximum Ag nanoparticles loading, exhibited higher BET surface area as compared to other samples. The larger surface area of nanodisk-like structures in AZ510 facilitates enhanced dye adsorption. In addition, Ag nanoparticles act as sinks for the photogenerated electrons and hence suppress the recombination of electrons and holes. The observed enhanced photocatalytic activity of Ag–ZnO hybrid plasmonic nanostructures is mainly due to citrate-assisted formation of nanodisks with better photocatalytic efficiency and improved sun-light utilization due to the plasmonic response of Ag nanoparticles, which suppress the recombination of photodegenerated electrons and holes.

## Conclusion

We have successfully synthesized the Ag–ZnO plasmonic nanohybrids with enhanced photocatalytic activity by a facile wet chemical method. ZnO nanostructures were decorated with Ag nanoparticles by citrate assisted photoreduction. Higher citrate concentrations resulted in the formation of nanodisks due to citrate-assisted oriented attachment of ZnO nanostructures. The photocatalytic efficiency of ZnO nanostructures has been found to increase with extent of Ag nanoparticles loading. We have demonstrated that the photocatalytic activity of ZnO nanostructures can be significantly enhanced upon decoration with Ag nanoparticles, which suppress the recombination of photodegenerated electrons and holes and improve sun-light utilization due to plasmonic response of Ag nanoparticles.

## Experimental

### Materials

Zinc nitrate hexahydrate (Zn(NO_3_)·6H_2_O, Merck, Germany) and potassium hydroxide (KOH, SRL, India) were used as the starting materials for the synthesis of ZnO nanostructures. Silver nitrate (AgNO_3_, Spectrochem, India) and trisodium citrate (Na_3_C_6_H_5_O_7_, CDH, India) were used for the photodeposition of Ag nanoparticles onto ZnO nanostructures. Methylene blue (MB, SRL India) was used as dye for photocatalysis studies. All chemicals were of analytical grade and were used as received without any further purification.

### Synthesis of ZnO nanoparticles

ZnO nanoparticles were prepared by a water-based facile wet chemical method by slowly adding an aqueous KOH solution to a Zn(NO_3_)·6H_2_O solution at room temperature under stirring. In a typical synthesis, 200 mL aqueous solution of 0.1 M Zn(NO_3_)·6H_2_O and 100 mL of 2 M KOH solution were prepared and separately stirred until they became clear. The KOH solution was then added drop-wise into the Zn salt solution under continuous stirring so as to reach pH ≈ 12. The mixture with white precipitates was continuously stirred for 2 h and aged overnight at room temperature. The precipitate was then filtered out, thoroughly washed with deionized water and then dried in an oven for 20 h at 80 °C leading to the formation of ZnO nanoparticles in powder form.

### Synthesis of Ag–ZnO hybrid nanostructures

For the synthesis of Ag–ZnO hybrid nanostructures, synthesized ZnO nanoparticles were redispersed in 100 mL of deionized water under sonication. To this aqueous solution, different concentrations of trisodium citrate ranging from 0.1 to 20 mM were added and continuously stirred overnight. Silver nitrate of concentrations varying from 0.1 to 2 mM was added into these solutions under stirring for 30 min in the dark. The [Ag^+^]/[citrate] concentration ratios in these solutions were chosen to be 1:1 and 1:10 for different AgNO_3_ concentrations. Photoreduction of Ag ions was carried out by irradiation of these suspensions with sun light for 2 h for the photodeposition of Ag nanoparticles onto the surface of ZnO nanostructures. The color of the suspensions changed rapidly from white to pale yellow and in some cases to grey depending on the Ag concentration. The colored precipitates formed were centrifuged, thoroughly washed with deionized water and dried in an oven at 80 °C for 20 h. The nomenclature of the synthesized samples obtained with different [Ag^+^]/[citrate] concentration ratios and AgNO_3_ concentrations are given in Table 1.

**Table 1:** Sample nomenclature and the estimated photocatalytic efficiency.

sample	experimental conditions		photocatalytic Efficiency η (%) (*t* = 20 min)
	AgNO_3_ concentration (mM)	[Ag^+^]/[citrate] ratio	

**PZ**	0	—	52.1
**AZ11**	0.1	1:1	66.3
**AZ21**	0.2		69.7
**AZ31**	0.5		75.3
**AZ210**	0.2	1:10	73.1
**AZ310**	0.5		78.7
**AZ410**	1		80.3
**AZ510**	2		94.1

### Characterization

The structural properties of the synthesized samples were determined by powder X-ray diffraction (XRD) at room temperature by using a Panalytical X’pert Pro diffractometer with Cu Kα radiation (λ = 0.1542 nm). Field emission scanning electron microscopy (FESEM) was used for studying the morphology of ZnO and Ag–ZnO nanostructures. Transmission electron microscopy (TEM) investigations were carried out using a FEI, TECNAI G^2^ F30, S-TWIN microscope operating at 300 kV. TEM machine is equipped with an Orius CCD camera from Gatan Inc., a HAADF detector from Fischione (Model 3000), an EDS detector from EDAX Inc., and a post-column Imaging Filter (Quantum SE, Model 963) from Gatan Inc. The sample was dispersed in ethanol by using an ultrasonic bath, mounted on a carbon coated Cu grid, dried, and used for TEM measurements. The optical properties of the samples were studied by UV–visible absorption spectroscopy and photoluminescence (PL) spectroscopy at room temperature. The powder samples were dispersed in deionized water by sonication and their optical properties were studied by UV–visible absorption spectroscopy by using a dual beam spectrophotometer HITACHI U3300 in the wavelength range of 200–800 nm, with deionized water as the reference medium. PL studies using excitation at 325 nm were carried out on samples coated onto Si substrates. The surface area of selected samples was determined by N_2_ adsorption/desorption measurements by using a BET 2375 surface area analyzer.

### Photocatalytic measurements

The photocatalytic activity of ZnO nanostructures and Ag–ZnO hybrid plasmonic nanostructures was evaluated by the degradation of methylene blue (MB) dye under sun-light irradiation. For the photocatalytic studies, typically 5 mg of as-synthesized ZnO and Ag–ZnO nanostructures were ultrasonically dispersed in 5 mL deionized water. Aqueous MB solution was added to the photocatalyst mixture and thoroughly mixed. The reaction suspensions containing 10 μM MB and different (ZnO, Ag–ZnO) photocatalysts were irradiated with sun light for different times (10, 20, 40 min) with intermittent shaking for uniform mixing of the photocatalysts with the MB solution. The photocatalysts were removed from the suspensions by centrifugation following the sun light exposure. The concentration of MB in the resultant solutions were monitored by UV–visible absorption spectroscopy studies in the wavelength range of 200–800 nm, with deionized water as the reference medium. The photocatalytic degradation efficiency of the photocatalysts for MB dye was calculated using the following formula:


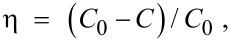


where *C*_0_ is the concentration of aqueous MB solution before addition of any photocatalyst and *C* is the concentration of MB in the reaction suspension with photocatalyst following sun-light exposure for time *t*.
